# High Concentrations of β_2_‐Microglobulin Do Not Inhibit In Vitro Generation of Functional Dendritic Cells

**DOI:** 10.1155/jimr/2924555

**Published:** 2026-01-05

**Authors:** Pavla Taborska, Dmitry Stakheev, Katerina Krausova, Jirina Bartunkova, Daniel Smrz

**Affiliations:** ^1^ Department of Immunology, Second Faculty of Medicine, Charles University and Motol University Hospital, Prague, Czech Republic, cuni.cz

**Keywords:** β_2_-microglobulin, dendritic cells, T cells

## Abstract

β_2_‐microglobulin (β2M) is a small protein playing a critical role in stabilizing major histocompatibility complex class I (MHC‐I) molecules on nucleated cells. Elevated levels of β2M have been observed in several cancers, inflammatory and autoimmune conditions, and renal failures. High concentrations of β2M were reported to inhibit in vitro generation of functional dendritic cells (DCs). However, our findings showed that β2M exerts a negative effect on DCs only when contaminated with endotoxins. We found that β2M preparations with a high level of endotoxin impurities matured DCs, but that this effect was not seen with functional β2M preparations with low levels of endotoxin impurities, thus showing the maturation effect was due to endotoxin stimulation. We confirmed that the high‐level endotoxin β2M compromised the in vitro differentiation of monocytes into DCs. In contrast, a low‐level endotoxin β2M had no negative impact on DC differentiation nor prevented their maturation and functionality. Moreover, regardless of the levels of endotoxin impurities, β2M stabilized the expression of MHC‐I molecules, confirming its functionality in the experimental settings. Our results show that β2M does not compromise the differentiation of DCs and indicate that elevated levels of β2M are unlikely to negatively regulate the immune system. These results have significant implications for understanding the functions of high β2M concentrations in clinical contexts and in vitro applications.

## 1. Introduction

β_2_‐microglobulin (β2M) is a nonglycosylated polypeptide that serves as the invariant chain of major histocompatibility complex class I (MHC‐I) molecules, which are critical for antigen presentation [1, 2]. β2M is expressed by all nucleated cells, and its serum levels in adults are often found between 1 and 3 µg/mL [3, 4]. Decreased levels of β2M can occur in certain genetic MHC‐I deficiencies or immunodeficiencies [2]. Conversely, increased levels can be the consequence of renal dysfunction or chronic kidney disease [5]. Elevated β2M levels are also associated with autoimmune diseases, viral infections, hematologic malignancies, and solid tumors [6–10]. These elevated levels are presumably caused by immune activation, which leads to greater turnover of MHC‐I molecules, as well as increased synthesis and shedding of β2M [3, 8, 11]. Despite its important physiological role, there is evidence suggesting that β2M can regulate the immune system. Elevated serum and plasma levels of β2M have been observed in various pathologies to correlate with immune cell activation [1]. Conversely, Xie et al. [12] reported that high concentrations of β2M, exceeding 10 µg/mL, inhibit in vitro generation of functional monocyte‐derived dendritic cells (moDCs). moDCs differentiated in the presence of β2M displayed an immunosuppressive phenotype characterized by high production of interleukin 6 (IL‐6), IL‐8, and IL‐10, while showing significantly lower expression of CD83, HLA‐ABC, costimulatory molecules, and adhesion molecules. Additionally, β2M has been found to activate the STAT3 signaling pathway, leading to the production of moDCs that are functionally less effective in stimulating lymphocytes [12]. Since β2M is frequently used in clinical diagnostics as well as in various in vitro experimental preparations and cell production, we further investigated its immunomodulatory effects during different phases of moDC preparation from peripheral blood mononuclear cells (PBMCs).

## 2. Materials and Methods

### 2.1. Specimen and β2M

The source material for DC preparation was cryopreserved PBMCs that were isolated from buffy coats from healthy blood donor volunteers, as described previously [13]. The buffy coats from the volunteers were obtained from the Institute of Hematology and Blood Transfusion in Prague. Each volunteer signed informed consent for using its blood‐derived products for future research. The research involving human specimens was approved in accordance with the ethical standards of the institutional research committee—the Ethics Committee of the Motol University Hospital in Prague (protocol code: EK–753.1.8/21), and all experiments were performed in compliance with the 1964 Helsinki Declaration and its later amendments or comparable ethical standards. The β2M was from three suppliers: Sigma (Saint Louis, MO, USA), MP Biologicals (Solon, OH, USA), and Abcam (Cambridge, United Kingdom). The Sigma β2M was from human urine, catalog number SAE0112‐1000UG; the MP Biologicals β2M was also from human urine, catalog number 770953; and the Abcam β2M was a recombinant protein, catalog number ab151631.

### 2.2. Preparation of moDCs, Their Maturation, and Evaluation of Their Phenotype and Functionality

MoDC were differentiated from PBMCs for 5 days using GM‐CSF and IL‐4 (1000 IU/mL) according to the protocol described previously [13] (Figure 1A). During the 5‐day differentiation, the cell culture medium was supplemented, or not, with 10 µg/mL of β2M on days 0 and 4 of the cell differentiation. On day 5, the differentiated cells were harvested and matured overnight (18–24 h) with indicated concentrations of β2M or 10 μg/mL of the TLR7/8 agonist R‐848 (Enzo Life Sciences, Farmingdale, NY, USA) with or without 200 μg/mL anti‐TNFα monoclonal antibody (Adalimumab, Selleck Chemicals, Houston, TX, USA), and the matured cells were analyzed by flow cytometry for the surface expression of the maturation markers CD80, CD83, CD86, and HLA‐DR as detailed [13]. Alternatively, moDC were differentiated/matured from PBMCs for 2 days (48 h) using GM‐CSF and IL‐4 (1000 IU/mL) in the presence or absence of 10 µg/mL of Sigma β2M or 10 μg/mL of R‐848. The moDC functionality was determined through the evaluation of the DC‐induced stimulation of autologous lymphocytes and Ki‐67 intracellular staining, as detailed [14].

### 2.3. Evaluation of β2M Purity and Functionality

The endotoxin levels were determined by the GMP Control Laboratory, ALS Czech Republic, s.r.o. (Prague, Czech Republic) using a certified turbidimetric‐based LAL. The results were approved and authorized for reporting according to the procedures specified in the document CZ_SOP_D06_GMP_02 Processing of GMP Project, which is in accordance with the procedures specified in the FDA document CFR Title 21, Part 11. T2 cells (AATC, Manassas, VA, USA) and their HLA‐A2 surface expression were used to evaluate β2M functionality [15, 16].

### 2.4. Evaluation of β2M Functionality

T2 cells were cultured in a fetal bovine serum (FBS)‐containing medium (KM medium; RPMI 1640 medium [Thermo Scientific, Waltham, MA, USA], 10% FBS [HyClone, GE Healthcare Life Sciences, South Logan, UT, USA], 100 U/mL penicillin‐streptomycin, and 2 mM GlutaMax [Thermo Scientific]). To perform the evaluation, the cultured T2 cells were extensively rinsed with KM medium without FBS to remove FBS from the cells. Then 100 μL of T‐cell suspension (1 × 10^6^ cells/mL) was transferred to a U‐bottom 96‐well plate wells (Nalgene, Rochester, NY, USA) and supplemented, or not, with 20 µg/mL of β2M and 2 µg/mL of human PSA pooled peptides (PepMix; cat.# PM‐PSA, JPT Peptide Technologies, Berlin, Germany). After overnight incubation (37°C, 5% CO2, 18–24 h), the cell suspension was transferred into V‐bottom 96‐well plate well (Nalgene), rinsed with ice‐cold PBS supplemented with 2 mM EDTA (PBS/EDTA), and stained with the Alexa Fluor 647‐labeled HLA‐A2‐specific antibody (BioRad, Hercules, CA, USA). The cells were rinsed with PBS/EDTA, supplemented DAPI (100 ng/mL, Thermo Scientific), and immediately analyzed using FACSAria II or BD LSRFortessa flow cytometer (Becton Dickinson, Franklin Lakes, NJ, USA), and the acquired data were analyzed with FlowJo software (Tree Star, Ashland, OR, USA).

### 2.5. Statistical Analysis

The values were calculated based on the sample size using GraphPad Prism 10 (GraphPad Software, La Jolla, CA, USA). Statistical significance ( ^∗^ *p* < 0.05,  ^∗∗^ *p* < 0.01,  ^∗∗∗^ *p* < 0.005,  ^∗∗∗∗^ *p* < 0.0001) among variables was determined using the paired one‐way ANOVA with Tukey’s posttest.

## 3. Results and Discussion

### 3.1. β_2_M Preparations Can Differentially Mature moDCs

A previous study has shown that high concentrations of β2M, exceeding 10 µg/mL, impair the differentiation of monocytes into moDCs [12]. Similar effects have also been reported for compounds that elicit DC maturation, such as TLR ligands [17] or agonists [18]. While β2M is frequently used in vitro for various experimental and cell production applications that require stabilizing MHC‐I expression on the cell surface [15, 16], we hypothesized that high concentrations of β2M, like maturation compounds, may also contribute to DC maturation. Using urine‐isolated β2M from Sigma, we observed that concentrations at 10 µg/mL and higher induced maturation of moDCs, as indicated by increased surface expression of DC maturation markers CD80, CD83, CD86, and HLA‐DR (Figure 1B–D). This enhancement in marker expression is also known to be promoted by TNFα, which is produced upon DC maturation [19]. We found that this enhancement was inhibited by anti‐TNFα antibody (Adalimumab) in both β2M‐ and TLR7/8 agonist (R‐848)‐matured DCs (Figure 1E), suggesting that the mechanisms of DC maturation were similar. However, our attempts to replicate this maturation using recombinant β2M from Abcam (Figure 1F) or urine‐isolated β2M from MP Biologicals (Figure 1G) were unsuccessful.

Figure 1The impact of β2M on maturation of moDCs. (A) The design of the experiment: Sigma β2M (β2MS), Abcam recombinant β2M (β2Mrec), MP Biologicals β2M (β2MMPB), TLR7/8 agonist (R‐848). (B) The gating strategy of flow cytometry data of matured moDCs. (C) Histograms of the cell surface staining of DC maturation marker CD83 in moDCs (CD11c^+^ cells) matured with vehicle, the indicated concentrations of Sigma β2M (β2MS), or 10 μg/mL of R‐848. (D) The staining intensities of CD80, CD83, CD86, and HLA‐DR in moDCs (CD11c^+^ cells) matured as in (C). (E) The staining intensities of CD80, CD83, CD86, and HLA‐DR in moDCs (CD11c^+^ cells) matured with vehicle, 10 μg/mL of Sigma β2M alone (β2MS) or in the presence of 200 μg/mL Adalimumab (β2MS/Ada), or 10 μg/mL of R‐848 alone (R‐848) or in the presence of 200 μg/mL Adalimumab (R‐848/Ada). (F) The staining intensities of CD83 and CD86 in moDCs (CD11c^+^ cells) matured with vehicle, 10 μg/mL of Sigma β2M (β2MS), 10 μg/mL of Abcam recombinant β2M (β2Mrec), or 10 μg/mL of R‐848. (G) The staining intensities of CD83 and CD86 in moDCs (CD11c^+^ cells) matured with vehicle, 10 μg/mL of MP Biologicals β2M (β2MMPB), or 10 μg/mL of R‐848. In (B) and (C), representative dot plots and histograms are shown. In (D–G), box and whisker plots (2.5–97.5 percentile) are shown, and statistical significances of differences among the tested groups are indicated ( ^∗^ *p*  < 0.05,  ^∗∗^ *p*  < 0.01,  ^∗∗∗^ *p* < 0.001,  ^∗∗∗∗^ *p* < 0.0001; (D, E) *n* = 5 donors, (F, G) *n* = 4 donors; paired one‐way ANOVA with Tukey’s posttest). In (A), the figure was created with Biorender (ref. no. KS292SEB2L).

**Figure:**
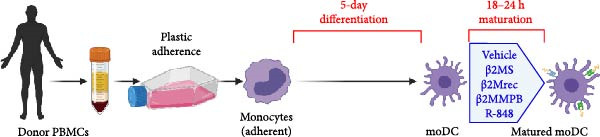
(A)

**Figure:**
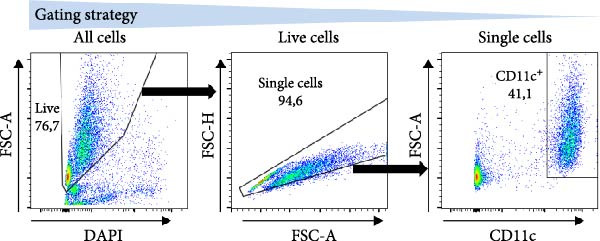
(B)

**Figure:**
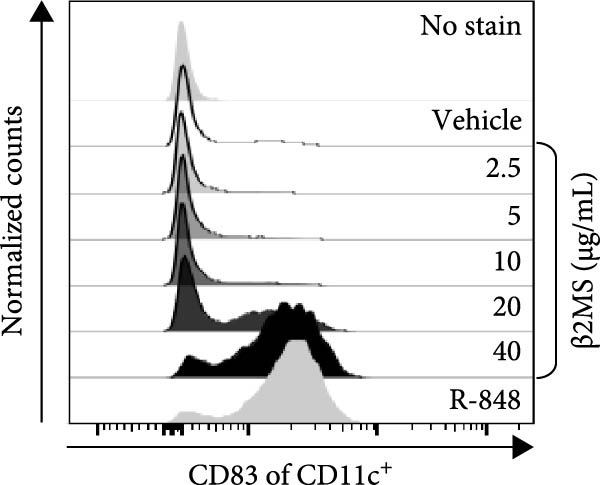
(C)

**Figure:**
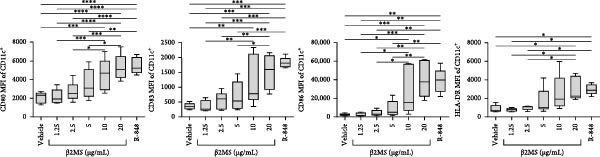
(D)

**Figure:**
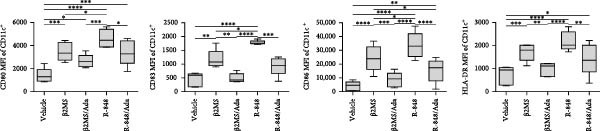
(E)

**Figure:**
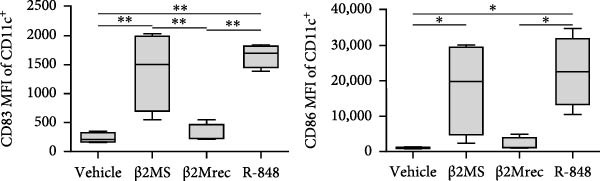
(F)

**Figure:**
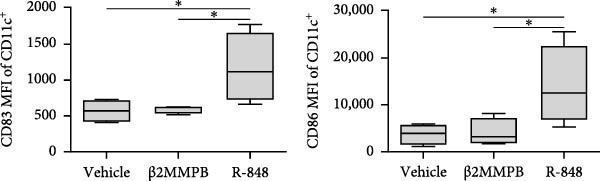
(G)

### 3.2. β2M With a Low Level of Endotoxin Impurities Does Not Mature moDCs

The differences in the ability of β2M to mature moDCs suggested that various β2M preparations could contain impurities that influenced DC maturation. Conversely, it was also possible that some preparations of β2M had impaired functionality. To investigate the former, we analyzed the endotoxin levels in two urine isolates of β2M: the DC‐maturing preparation from Sigma and the nonmaturing preparation from MP Biologicals, using the limulus amebocyte lysate (LAL) method in a certified laboratory.

The analysis revealed that the endotoxin levels in the DC‐maturing β2M from Sigma were 2110 EU/mL. This preparation had a concentration of 0.393 mg/mL, and when diluted to 10 µg/mL, it still contained endotoxin levels of 53 EU/mL. These findings indicated that the observed effects of the high β2M concentration in Figure 1A–F were likely due to these impurities rather than the β2M itself [20–23]. In contrast, the endotoxin levels in the DC nonmaturing β2M from MP Biologicals were below 2.5 EU/mL. This preparation had a concentration of 1 mg/mL, and when diluted to 10 µg/mL, the endotoxin levels decreased to below 0.025 EU/mL. Therefore, the inability of this β2M preparation to mature DCs (as shown in Figure 1G) was presumably due to these extremely low endotoxin levels during the DC maturation process.

### 3.3. β2M With a Low Level of Endotoxin Impurities Is Functional

The correlation between the presence or absence of endotoxins in β2M preparations and their negative impact on DC differentiation and functionality indicated that endotoxin impurities were responsible for this effect, as previously reported [17]. However, to confirm that the functionality of β2M was not contributing to this negative effect, we needed to demonstrate that the low‐endotoxin β2M was functional and capable of stabilizing the expression of MHC‐I on the cell surface [15]. To test this, we utilized the T2 cell line, which expresses the HLA‐A2 variant of MHC‐I on its surface, the expression of which is enhanced in the presence of extracellular β2M and extracellular peptides [16]. As shown in Figure 2A–C, regardless of whether Sigma (high‐endotoxin) or MP Biologicals (low‐endotoxin) β2M preparations were used, both preparations increased the expression of HLA‐A2 on the cell surface in the presence of extracellular prostate‐specific antigen (PSA) peptides. In the case of the high‐endotoxin β2M, the expression was presumably higher, as DC maturation is also associated with increased MHC‐I expression on their surface [24, 25].

Figure 2The β2M functionality and the impact of β2M on monocyte differentiation into moDCs and evaluation of moDC functionality. (A) The gating strategy of flow cytometry data of T2 cells. (B) Representative histograms of the cell surface staining of HLA‐A2 in T2 cells incubated with vehicle, 2 μg/mL of PSA PepMix (PSA), or 2 μg/mL of PSA PepMix (PSA) with 10 μg/mL of high‐endotoxin Sigma β2M (PSA/β2MS) or low‐endotoxin MP Biologicals β2M (PSA/β2MMPB). (C) The evaluated data in (B). (D) The design of the experiment. (E, F) Monocytes from PBMCs were differentiated for 5 days into moDCs with GM‐CSF and IL‐4 in the absence (vehicle) or presence of 10 μg/mL of high‐endotoxin Sigma β2M (β2MS) or low‐endotoxin MP Biologicals β2M (β2MMPB) or TLR7/8 agonist (R‐848). The differentiated cells were then matured with 10 μg/mL of R‐848. In (E), the viability (E1) and the proportions of matured moDCs (CD11c^+^) (E2) are shown. In (F), the cell surface staining of DC maturation marker CD83 in matured moDCs (CD11c^+^ cells) is shown. (G, H) Monocytes from PBMCs were differentiated/matured for 2 days into moDCs with GM‐CSF and IL‐4 in the absence (vehicle) or presence of 10 μg/mL of high‐endotoxin Sigma β2M (β2MS) or R‐848. In (G), the viability (G1) and the proportion of moDCs (CD11c^+^) (G2) are shown. In (H), the cell surface staining of DC maturation marker CD83 in 2‐day moDCs (CD11c^+^ cells) is shown. (I–K) The moDCs differentiated and matured in (E, F) were used to stimulate autologous lymphocytes at 1:4 ratio (DCs:lymphocytes) for 5 days, and Ki‐67 frequencies in CD4^+^ and CD8^+^ T cells were determined. (I) The gating strategy of flow cytometry data of DC‐expanded lymphocytes. (J) Representative dot plots of Ki‐67 frequencies in CD4^+^ T cells (J1) or CD8^+^ T cells (J2) stimulated with matured moDCs. (K) The evaluated frequencies of Ki‐67 in CD4^+^ and CD8^+^ T cells in (J). In (A, B, I, and J), representative dot plots and histograms are shown. In (C, E–H, and K), box and whisker plots (2.5–97.5 percentile) are shown, and statistical significances of differences among the tested groups are indicated ( ^∗^ *p* < 0.05,  ^∗∗^ *p* < 0.01,  ^∗∗∗^ *p* < 0.001,  ^∗∗∗∗^ *p* < 0.0001; (C) *n* = 7 independent experiments, (E) *n* = 7 donors, (F) *n* = 3 donors, (G, H) *n* = 4 donors, (K) *n* = 7 donors; paired one‐way ANOVA with Tukey’s posttest). In (D), the figure was created with Biorender (ref. no. UR292SEI3K).

**Figure:**
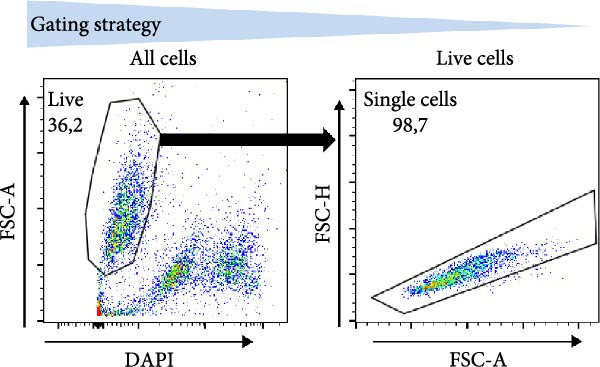
(A)

**Figure:**
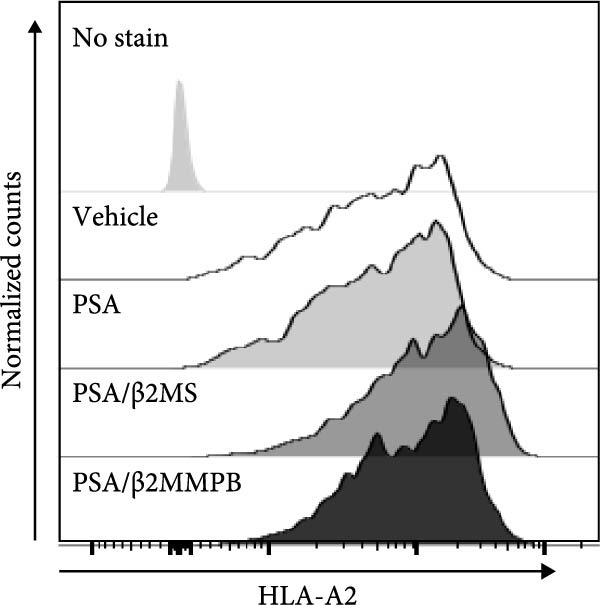
(B)

**Figure:**
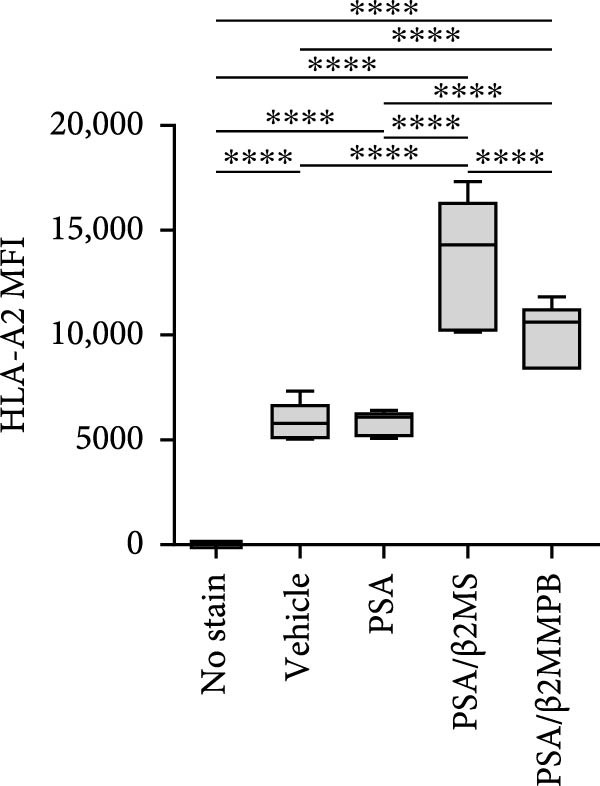
(C)

**Figure:**
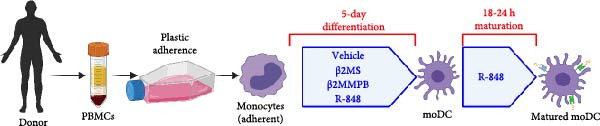
(D)

**Figure:**
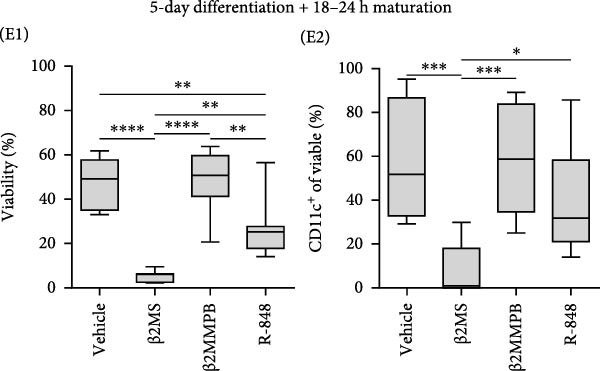
(E)

**Figure:**
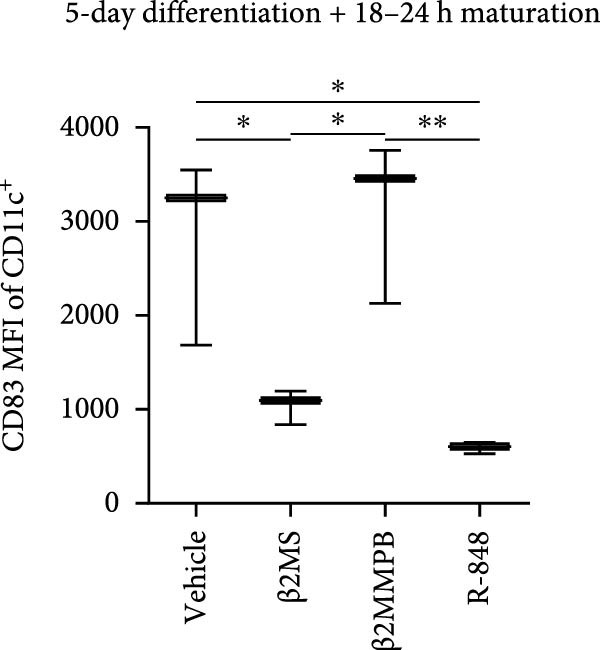
(F)

**Figure:**
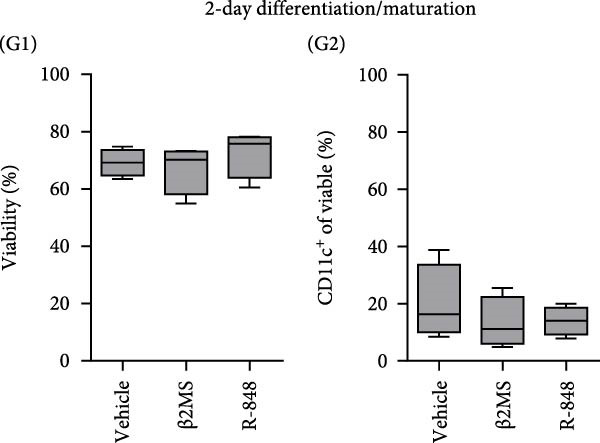
(G)

**Figure:**
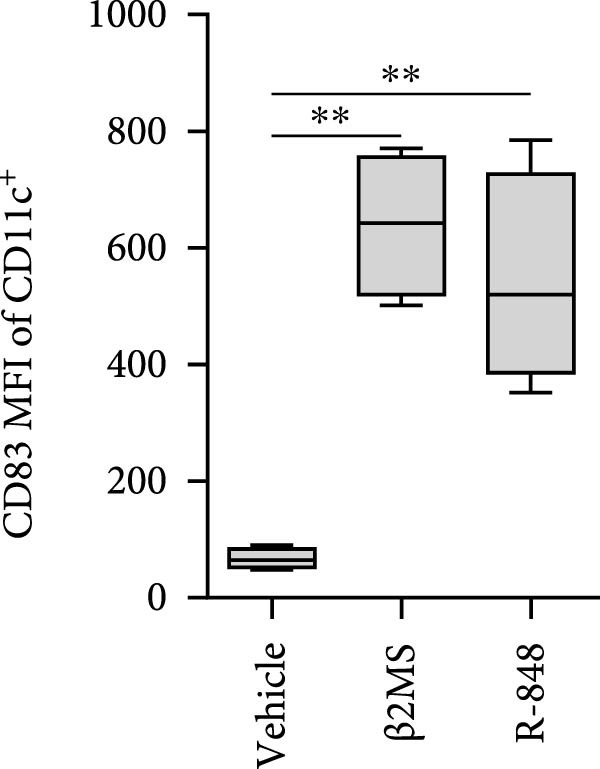
(H)

**Figure:**
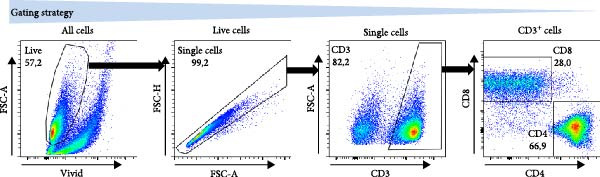
(I)

**Figure:**
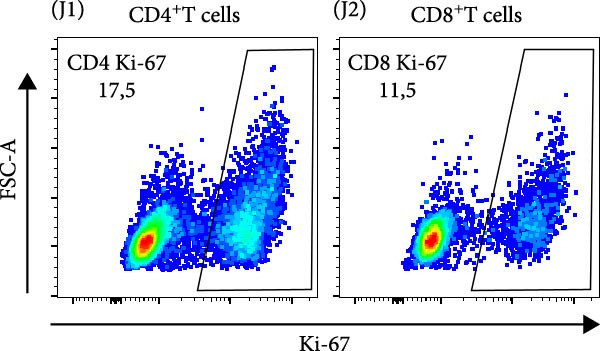
(J)

**Figure:**
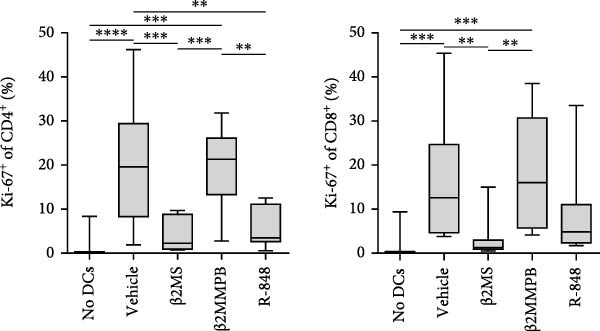
(K)

### 3.4. β2M With a Low Level of Endotoxin Impurities Does Not Compromise the Differentiation of Monocytes Into moDCs

Our findings so far demonstrated that a functional, low‐level endotoxin β2M was unable to mature moDCs. Since endotoxins can also block the potential of monocytes to differentiate into moDCs [17], we next investigated whether the functional, low‐level endotoxin β2M could also recapitulate previous findings of Xie et al. [12], which showed that high concentrations of β2M inhibit in vitro generation of functional moDCs. We, therefore, conducted a parallel test to examine how two different preparations of β2M—one from Sigma (high‐endotoxin) and the other from MP Biologicals (low‐endotoxin)—affected 5‐day differentiation of moDCs from monocytes and their subsequent maturation (Figure 2D). For comparison, we used R‐848 as a positive control during the 5‐day differentiation since this TLR7/8 agonist has also been shown to impair DC differentiation [18]. As shown in Figure 2E, the presence of R‐848 significantly inhibited the 5‐day differentiation of monocytes into moDCs. We observed that 5‐day‐differentiated cells in the presence of R‐848 and those also subsequently matured with R‐848 for 18–24 h showed reduced viability and a lower proportion of the moDC population (CD11c^+^), thereby confirming earlier findings [18]. Furthermore, maturation with R‐848 resulted in minimal expression of the moDC maturation marker CD83 (Figure 2F). Surprisingly, we found that the high‐endotoxin β2M from Sigma had an even greater negative effect on DC differentiation than R‐848 (Figure 2E), corroborating a previous report [12]. Similar to R‐848, the high‐endotoxin β2M negatively affected the surface expression of CD83 after subsequent maturation with R‐848 (Figure 2F), which also corroborated the findings of the previous report [12]. In contrast, however, the low‐endotoxin β2M from MP Biologicals did not affect DC differentiation nor CD83 expression compared to the control group (Figure 2E,F).

Previous studies have demonstrated that LPS and other maturation agents can be used for rapid DC generation from monocytes [26, 27]. The phenotype we observed in 5‐day differentiated and 1‐day (18–24 h) matured moDCs could therefore be explained by endotoxin‐mediated overactivation and subsequent cell death. To address this, we employed a rapid 2‐day DC‐differentiation protocol using GM‐CSF and IL‐4, in the presence of 10 µg/mL of high‐endotoxin β2M from Sigma or R‐848. As shown in Figure 2G,H, a 2‐day exposure to either high‐endotoxin β2M or R‐848 did not reduce cell viability or decrease the proportion of moDCs (CD11c^+^) among the differentiated cells. Moreover, both compounds induced comparable upregulation of the maturation marker CD83. These results indicate that the adverse effects of high‐endotoxin β2M or R‐848 on moDCs are not manifested under conditions of rapid DC generation [26, 27]. However, this accelerated approach still does not resolve the underlying issue of endotoxin contamination during DC production for clinical applications.

### 3.5. The Functionality of moDCs Differentiated in the Presence of β2M With a Low Level of Endotoxin Impurities is Not Compromised

The discrepancies between the two β2M preparations in the ability to impact the differentiation of monocytes into moDCs indicated that these discrepancies could also translate into the ability of the DCs to stimulate the proliferation of autologous T cells. As shown in Figure 2I–K, moDCs generated in the presence of R‐848 or the high‐endotoxin β2M had largely compromised their ability to stimulate the proliferation of CD4^+^ and CD8^+^ T cells. On the other hand, no adverse effects were found for moDCs differentiated in the presence of the low‐endotoxin β2M as compared to moDCs differentiated and matured in the absence of β2M. These data showed that a functional, low‐level endotoxin β2M was not able to negatively compromise the functionality of moDCs.

## 4. Concluding Remarks

Collectively, these data showed that a high concentration of functional β2M with low levels of endotoxins does not compromise DC differentiation and functionality. Therefore, these findings do not support conclusions from Xie et al. [12] that β2M has immunosuppressive activities. These results have significant implications for understanding the role of β2M levels in clinical settings as well as in vitro applications. Furthermore, they highlight the potential artifacts that endotoxin impurities may introduce in preclinical research.

## Conflicts of Interest

Jirina Bartunkova is a part‐time employee and a minority shareholder of Sotio Biotech, a.s., a biotech company developing immuno‐oncology therapies. The remaining authors declare no conflicts of interest.

## Funding

The research was supported by the Ministry of Health, Czech Republic—projects NU22‐03‐00300 and NU23‐08‐00071.

## Data Availability

Data are available upon request due to privacy/ethical restrictions.
